# Phylogenetic analysis, subcellular localization, and expression patterns of RPD3/HDA1 family histone deacetylases in plants

**DOI:** 10.1186/1471-2229-9-37

**Published:** 2009-03-28

**Authors:** Malona V Alinsug, Chun-Wei Yu, Keqiang Wu

**Affiliations:** 1 Institute of Plant Biology, College of Life Science, National Taiwan University, Taipei, Taiwan

## Abstract

**Background:**

Although histone deacetylases from model organisms have been previously identified, there is no clear basis for the classification of histone deacetylases under the RPD3/HDA1 superfamily, particularly on plants. Thus, this study aims to reconstruct a phylogenetic tree to determine evolutionary relationships between RPD3/HDA1 histone deacetylases from six different plants representing dicots with *Arabidopsis thaliana, Populus trichocarpa, and Pinus taeda*, monocots with *Oryza sativa *and *Zea mays*, and the lower plants with *Physcomitrella patens*.

**Results:**

Sixty two histone deacetylases of RPD3/HDA1 family from the six plant species were phylogenetically analyzed to determine corresponding orthologues. Three clusters were formed separating Class I, Class II, and Class IV. We have confirmed lower and higher plant orthologues for AtHDA8 and AtHDA14, classifying both genes as Class II histone deacetylases in addition to AtHDA5, AtHDA15, and AtHDA18. Since Class II histone deacetylases in other eukaryotes have been known to undergo nucleocytoplasmic transport, it remains unknown whether such functional regulation also happens in plants. Thus, bioinformatics studies using different programs and databases were conducted to predict their corresponding localization sites, nuclear export signal, nuclear localization signal, as well as expression patterns. We also found new conserved domains in most of the RPD3/HDA1 histone deacetylases which were similarly conserved in its corresponding orthologues. Assessing gene expression patterns using Genevestigator, it appears that RPD3/HDA1 histone deacetylases are expressed all throughout the plant parts and developmental stages of the plant.

**Conclusion:**

The RPD3/HDA1 histone deacetylase family in plants is divided into three distinct groups namely, Class I, Class II, and Class IV suggesting functional diversification. Class II comprises not only AtHDA5, AtHDA15, and AtHDA18 but also includes AtHDA8 and AtHDA14. New conserved domains have also been identified in most of the RPD3/HDA1 family indicating further versatile roles other than histone deacetylation.

## Background

Histone deacetylases (HDACs or HDAs) have been generally responsible for the deacetylation of lysine residues on the N-terminal region of core histones. This usually gives a tag for epigenetic repression and plays an important role in transcriptional regulation, cell cycle progression and developmental events. They are classified into three different families namely the RPD3/HDA1 superfamily, sirtuin family, and the HD2 family [1]. Members of RPD3/HDA1 superfamily and sirtuin family are proteins homologous to the yeast reduced potassium dependency 3 (RPD3)/Hda1 and silent information regulator 2 (Sir2), respectively, whereas HD2 family is unique in plants. Phylogenetic studies classify the RPD3/HDA1 superfamily (non-sirtuin) histone deacetylases into three classes: the class 1 (RPD3-like), class 2 (HDA1-like), and an additional class defined by the human HsHDA511 (also called HsHDAC11) [2]. This third class has been named class 4 to distinguish it from the unrelated NAD-dependent sirtuin deacetylases, which have sometimes been called class 3 HDACs.

Among the RPD3/HDA1 superfamily histone deacetylases in Arabidopsis, AtHDA6, AtHDA19, and AtHDA18 have been demonstrated to play essential roles in plant development and environmental stress response [3-9]. Aside from regulating rRNA genes and affecting transgene expression and DNA methylation, AtHDA6 is also known as a global repressor involved in JA pathway, senescence, flowering, repression of embryonic properties, and establishment of nucleolar dominance [8,10-14]. Similarly, AtHDA19 is a global repressor in embryonic and flower development, JA and ethylene signaling, and regulates plant basal response via interaction with WRKY transcription factors [3-5,8,15,16]. Studies by Xu et al. [17] suggested the involvement of HDA18 in root epidermal patterning.

To date, there are 18 histone deacetylases known in Arabidopsis with the RPD3/HDA1 superfamily subdivided into three classes [1]. Four proteins were reported to be under Class I namely AtHDA6, AtHDA7, AtHDA9, and AtHDA19 while three have been shown to be classified under Class II, namely AtHDA5, AtHDA15, and AtHDA18. On the other hand, AtHDA2 was the sole plant histone deacetylase under the Class III group with no other identified plant homologue [1]. Still, there are those that remain unclassified but are grouped within the RPD3/HDA1 superfamily such as AtHDA8, AtHDA10, AtHDA14, and AtHDA17 [1,18]. On another study conducted by Fu *et al. *[19], results on the phylogenetic analysis of the RPD3/HDA1 histone deacetylases on *Oryza sativa *suggested this group to be divided into four classes. Due to the limited number of studies and incongruent results using different plant samples, there remains to be no clear basis for the classification of histone deacetylases under the RPD3/HDA1 superfamily, particularly on plants.

This study aims to reconstruct a phylogenetic tree to determine evolutionary relationships between RPD3/HDA1 histone deacetylases from six different plants representing dicots with *Arabidopsis thaliana, Populus trichocarpa, and Pinus taeda*, monocots with *Oryza sativa *and *Zea mays*, and the lower plants with the moss, *Physcomitrella patens*. Phylogenetic analyses of these 62 genes predicted to be members of the RPD3/HDA1 family showed that this superfamily constitute 3 distinct phylogenetic groups classified as Class I, Class II, and Class IV identifying corresponding orthologues in all the six plant species studied. AtHDA2 was classified as Class IV based on phylogenetic analyses and sequence similarity to its mammalian orthologue, hsHDA511, and to distinguish it from sirtuin deacetylases.

## Results

### Phylogenetic analyses of RPD3/HDA1 histone deacetylases in Arabidopsis, yeast, and metazoans

**Figure 1:**
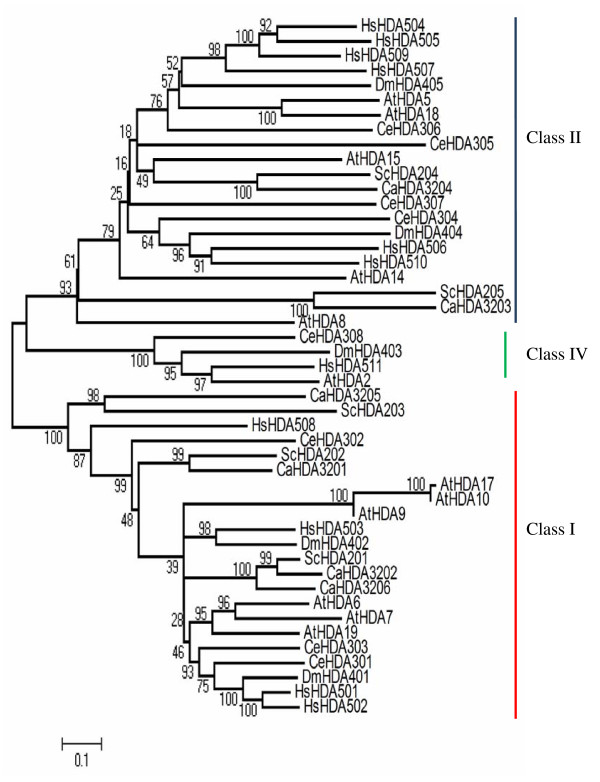
**Bootstrap consensus tree of RPD3/HDA1 family histone deacetylases in Arabidopsis, yeast and other metazoans**. Neighbor Joining phylogenetic tree of RPD3/HDA1 type histone deacetylases in Arabidopsis (AtHDA2-18), yeast (ScHDA201-205), and other metazoans including *Caenorhabditis elegans *(CeHDA301-308), *Drosophila melanogaster *(DmHDA401-405), *Candida albicans *(CaHDA3201-3206), and *Homo sapiens *(HsHDA501-511) was reconstructed using MEGA4. Bootstrap support on the left of each node was inferred from 1000 replicates.

Prospective members of RPD3/HDA1 superfamily from six yeast and metazoan species (Table 1) including Arabidopsis were phylogenetically analyzed to determine evolutionary relationships and sequence homology (Figure 1). Based on the bootstrap consensus tree inferred from 1000 replicates, histone deacetylases similar to the yeast RPD3 classified as Class I includes ScHDA202, ScHDA201, ScHDA203 in *Saccharomyces cerevisiae*; CaHDA3201, CaHDA3202, CaHDA3205, and CaHDA3206 in *Candida albicans*; CeHDA301, CeHDA302, and CeHDA303 in *Caenorhabditis elegans*; DmHDA401 and DmHDA402 in *Drosophila melanogaster*; HsHDA501, HsHDA502, HsHDA503, and HsHDA508 in *Homo sapiens*; AtHDA6, AtHDA7, AtHDA19, AtHDA9, AtHDA10, AtHDA17 in *Arabidopsis thaliana*. On the other hand, Class II histone deacetylases manifesting high sequence similarity to the yeast Hda1 includes the following: ScHDA204 and ScHDA205 in *Saccharomyces cerevisiae*; CaHDA3204 and CaHDA3203 in *Candida albicans*; CeHDA304, CeHDA305, CeHDA306, and CeHDA307 in *Caenorhabditis elegans*; DmHDA404 and DmHDA405 in *Drosophila melanogaster*; HsHDA504, HsHDA505, HsHDA506, HsHDA507, HsHDA509, and HsHDA510 in *Homo sapiens*; while in *Arabidopsis thaliana*, AtHDA5, AtHDA18, AtHDA15, including AtHDA14 with 78% bootstrap support and AtHDA8 with 93% bootstraps all belong to the Class II group. Another cluster grouped as Class IV is only represented by the metazoans namely, CeHDA308, DmHDA403, HsHDA511, and AtHDA2. The emergence of the Class IV group from the metazoans may reflect evolutionary divergence paving the way for functional specialization in multicellular metabolic processes.

**Table 1:** RPD3/HDA1 superfamily histone deacetylases in yeast and metazoans used for phylogenetic analysis with *Arabidopsis thaliana*

	*Saccharomyces cerevisiae*	*Caenorhabditis elegans*	*Drosophila melanogaster*	*Homo sapiens*	*Candida albicans*
Class I	ScHDA201	CeHDA301	DmHDA401	HsHDA501	CaHDA3201
	(RPD3)	(hda-1)	(Rpd3)	(hsHDAC1)	CaHDA3202
	ScHDA202	CeHDA302	DmHDA402	HsHDA502	CaHDA3205
	(HOS2)	CeHDA303	(Hdac3)	(hsHDAC2)	CaHDA3206
	ScHDA203	(ceHDA-3)		HsHDA503	
	(HOS1)			(hsHDAC3)	
				HsHDA508	
				(hsHDAC8)	

Class II	ScHDA204	CeHDA304	DmHDA404	HsHDA504	CaHDA3203
	(HDA1)	CeHDA305	(HDAC6)	(hsHDAC4)	CaHDA3204
	ScHDA205	(ceHDA-5)	DmHDA405	HsHDA505	
	(HOS3)	CeHDA306	(HDAC4)	(hsHDAC5)	
		(hda-7)		HsHDA506	
		CeHDA307		(hsHDAC6)	
				HsHDA507	
				(hsHDAC7)	
				HsHDA509	
				(hsHDAC9)	
				HsHDA510	
				(hsHDAC10)	

Class IV	---	CeHDA308	DmHDA403	HsHDA511	---
			(CG31119)	(hsHDAC11)	

TOTAL:	5	8	5	11	6

*Alias and other names referred to the following histone deacetylases are written in parenthesis under its formal name assigned by chromatin database (ChromDB).

### Phylogenetic analyses of RPD3/HDA1 histone deacetylases in plants

**Figure 2:**
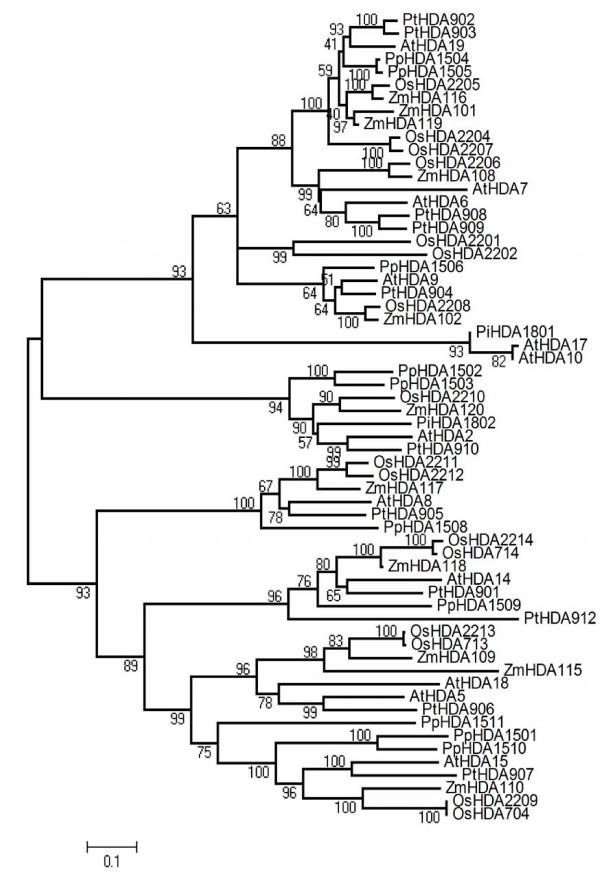
**A phylogenetic tree of RPD3/HDA1 histone deacetylases from *Arabidopsis thaliana, Populus trichocarpa, Pinus taeda, Oryza sativa, Zea mays*, and *Physcomitrella patens *was generated using the Neighbor Joining method**. The bootstrap consensus tree inferred from 1000 replicates is taken to represent the evolutionary history of the different RPD3/HDA1 proteins analyzed.

**Figure 3:**
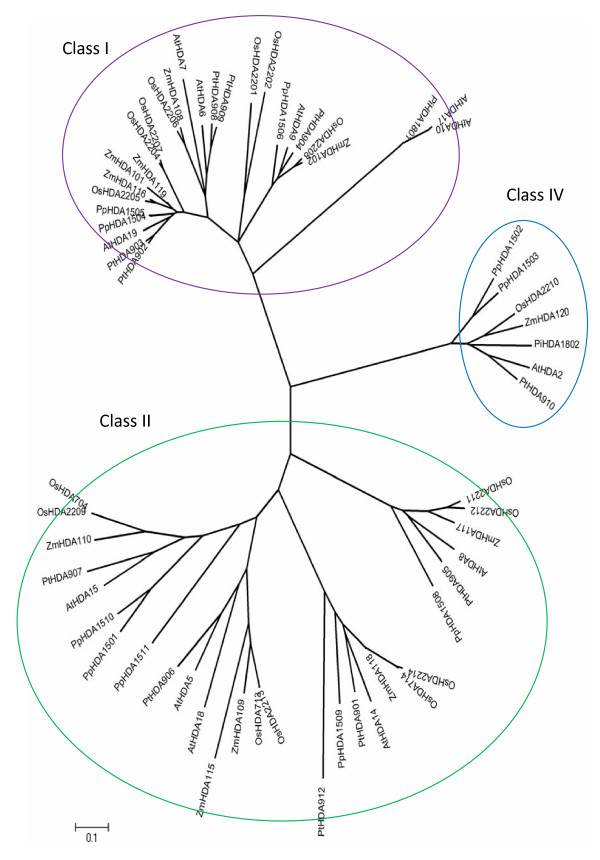
**Radiation tree**. Radiation tree of RPD3/HDA1 histone deacetylases in plants was inferred using the neighbor joining method.

To analyze further the classification of RPD3/HDA1 superfamily in plants, amino acid sequences from 62 RPD3/HDA1 proteins (Table 2) were used to derive sequence similarity and phylogenetic analyses. The overall phylogenetic tree inferred to represent all the 62 prospective RPD3/HDA1 proteins had a good bootstrap support indicating that the derived tree truly reflects the data used to generate it. As shown in Figure 2, a Neighbor Joining phylogenetic tree of RPD3/HDA1 histone deacetylases from *Arabidopsis thaliana, Populus trichocarpa, Pinus taeda, Oryza sativa, Zea mays*, and *Physcomitrella patens *shows the evolutionary relationships between these 62 proteins. As depicted in Figure 3, a radiation Neighbor Joining phylogenetic tree of RPD3/HDA1 histone deacetylases in the six plant species analyzed exhibited 3 different clades isolating the AtHDA2 group from the other groups. Thus, each clade can be classified as Class I for the RPD3 group, Class II for the HDA1-like group, and Class IV for the AtHDA2 group. All the RPD3/HDA1 proteins in Arabidopsis were distributed accordingly to their proposed class identifying further orthologues from other dicots, monocots, and in the lower plant moss.

**Table 2:** List of plant RPD3/HDA1 superfamily HDAs studied for phylogenetic analysis

				*Oryza sativa*			
						
	*Arabidopsis thaliana*	*Populus trichocarpa*	*Pinus taeda*	*indica*	*japon*.*	*Zea mays*	*Physcomitrella patens*
Class I	AtHDA7	PtHDA902	PiHDA1801	OsHDA2202	OsHDA701	ZmHDA116	PpHDA1504
	AtHDA9	PtHDA903		OsHDA2204	OsHDA702	ZmHDA102	PpHDA1505
	AtHDA10	PtHDA904		OsHDA2205	OsHDA703	ZmHDA101	PpHDA1506
	AtHDA17	PtHDA908		OsHDA2201	OsHDA705	ZmHDA119	
	AtHDA19	PtHDA909		OsHDA2206	OsHDA707	ZmHDA108	
				OsHDA2207	OsHDA709		
				OsHDA2208	OsHDA710		
					OsHDA711		

Class II	AtHDA5	PtHDA906		OsHDA2209	OsHDA704	ZmHDA109	PpHDA1501
	AtHDA8	PtHDA907		OsHDA2213	OsHDA713	ZmHDA110	PpHDA1510
	AtHDA14	PtHDA901		OsHDA2211	OsHDA714	ZmHDA115	PpHDA1511
	AtHDA15	PtHDA912		OsHDA2212		ZmHDA117	PpHDA1509
	AtHDA18	PtHDA905		OsHDA2214		ZmHDA118	PpHDA1508

Class IV	AtHDA2*	PtHDA910	PiHDA1802	OsHDA2210	OsHDA706	ZmHDA120	PpHDA1502
							PpHDA1503

Total	12	11	2	13	12	11	10

* RPD3/HDA1 family histone deacetylases from *Oryza sativa japonicum *were referenced from Fu *et al *(2007) where OsHDA704, OsHDA713, and OsHDA714 were included in the analyses in addition to the HDAs from *Oryza sativa indica*.

Moreover, Class II seems to manifest three different clusters separating the AtHDA8 group, AtHDA14 group, and the originally identified class II genes, AtHDA5, AtHDA15, and AtHDA18 [1]. In previous studies, AtHDA8 and AtHDA14 were considered unclassified proteins because they failed to cluster within the other HDAs even if they share the same conserved amino acid positions for Class II proteins. In addition, there were no known closely related proteins to compare with these HDAs to establish its classification as a Class II histone deacetylase [1]. Fortunately, we have identified representative monocot, other dicots, and a lower plant moss orthologues for all the Class II genes including AtHDA8 and AtHDA14 with high bootstrap support.

#### Class I: the RPD3-like Group

For the RPD3 group, it appears that Arabidopsis AtHDA10 and AtHDA17 are orthologues of the pine PtHDA1801 with a high bootstrap support of 93%. Although AtHDA10 and AtHDA17 are considered in-paralogues, its emergence after the species split technically makes it a *bona fide *orthologue [20]. However, the divergence of this group from the majority of Class I may be due to its short sequences but have high sequence similarity mainly in the RPD3 HDA domain exhibiting 93% bootstraps. Moving further, AtHDA9 of Arabidopsis was found to be similar with the poplar PtHDA904 but with a mediocre bootstrap of 51%. Its monocot counterparts OsHDA2208 and ZmHDA102 manifested a perfect bootstrap of 100%. Its lower plant orthologue PpHDA1506 from *Physcomitrella *diverged early on from its higher plant homologues exhibiting a 64% bootstrap. The poplar PtHDA909 and PtHDA908 appear to be orthologues of Arabidopsis AtHDA6 (80% bootstraps) which is highly similar still to AtHDA7 (64% bootstraps). With a 99% bootstraps as quantifier for its robustness, this dicot group found its monocot counterparts in OsHDA2206 and ZmHDA108 which were highly similar demonstrating 100% bootstrap support. On the other hand, AtHDA19 or AtHDA1 from Arabidopsis is orthologous to poplar PtHDA903 and PtHDA902 with 93% bootstraps. However, its immediate sister group in *Physcomitrella *PpHDA1504 and PpHDA1505 (with 100% bootstraps) showed low bootstrap support (41%) indicating that they may have the same ancestral roots but are unlikely to be the closest link. This gene group appears to be anomalous since *Physcomitrella *is expected to be the most ancestral which should have diverged first before any higher plant species split. It is quite strange to note that its monocot counterparts diverged twice earlier on during its evolutionary process. The more recent deviation lead to the speciation of rice OsHDA2205 and maize ZmHDA116 (100% bootstraps) and another monocot sister group ZmHDA101 and ZmHDA119 (97% bootstraps). Unfortunately, this two monocot sister groups have a low bootstrap support (40%) with which only one of this group can truly represent the closest monocot orthologues of AtHDA19. The earliest divergence, however, can be traced back to its rice orthologues coupled with gene duplications yielding OsHDA2204 and OsHDA2207 (100% bootstraps).

#### Class II: HDA1-like Group

Based on phylogenetic analyses as reflected on the consensus trees in Figure 2 and 3, the class II group is composed of three clusters wherein each cluster contains monocot, eu-dicot, and lower plant moss representatives. The first cluster is represented by the AtHDA8 group which diverged the earliest from the Class II group (93% bootstraps). The lower plant PpHDA1508 split earlier from the line (100% bootstraps) followed by the monocot/eu-dicot split with a mediocre 67% bootstraps. AtHDA8 from Arabidopsis appears to be orthologous to poplar PtHDA905 with 78% bootstraps. Its monocot sister group comprises ZmHDA117 from maize and OsHDA2211 and OsHDA2212 from rice (99% bootstraps) showing a perfect 100% bootstraps. The emergence of the second cluster made up of homologues of AtHDA14 seems to take a different evolutionary route from the classic lower plant/higher plant first followed by monocot/dicot split events. The poplar PtHDA912 diverged early from the line (96% bootstraps) followed by the divergence of the lower plant PpHDA1509 and the rest of higher plant group (76% bootstraps) separating further the dicots from the monocots (80% bootstraps). The dicot group was composed of Arabidopsis AtHDA14 which appears to be an orthologue of the poplar (65% bootstraps). Its monocot counterpart was basically comprised of the maize ZmHDA118 and *Oryza sativa *which underwent further speciation producing 2 orthologues from rice, OsHDA2214 and OsHDA714, both with 100% bootstrap support.

The third cluster is composed of the originally identified Class II genes, AtHDA5, AtHDA15, and AtHDA18 (99% bootstraps). Both AtHDA5 and AtHDA18 belong to the same group since AtHDA18 is basically a product of gene duplication of AtHDA5. From the dicot group, only PpHDA906 shows high sequence similarity to AtHDA5 (99% bootstraps). From its monocot counterpart, the maize ZmHDA115 diverged earlier prior to the separation of another maize, ZmHDA109, leading to the rice speciation of OsHDA2213 and OsHDA713, *indica *and *japonica*, respectively, with 100% bootstraps. Moreover, the lower plant moss HDA1511 (75% bootstraps) diverged from the main line followed by the lower plant moss HDA1501 and HDA1510 which shows high sequence similarity (100% bootstraps). This was followed by a dicot-monocot split (96% bootstraps) with the dicot represented by the poplar PtHDA907 which is an orthologue of Arabidopsis AtHDA15 (100% bootstraps). The monocot group, on the other hand, is represented by maize ZmHDA110 and rice which underwent further speciation in OsHDA2209 and OsHDA704, *indica *and *japonica*, respectively, both reflecting 100% bootstraps.

#### Class IV: HDA2 Group

Class IV group is represented accordingly by all the six plant species studied with Physcomitrella PpHDA1502 and PpHDA1503 (100% bootstraps) diverging early on from the higher plants reflecting a high 94% bootstraps. The monocot group deviated from the eu-dicot group (90% bootstraps) with rice OsHDA2210 and maize ZmHDA120 as the monocot orthologues. The eu-dicot group, however, underwent further speciation separating the gymnosperm PiHDA1802 from the angiosperms AtHDA2 and PtHDA910 (99% bootstraps), from Arabidopsis and poplar, respectively.

### Conserved Domains

**Figure 4:**
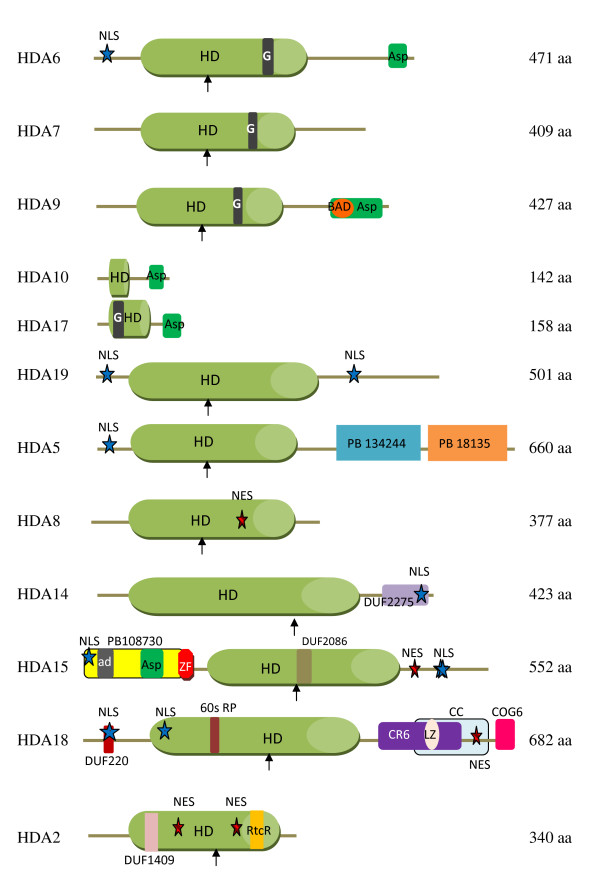
**Conserved domains of RPD3/HDA1 family histone deacetylases in Arabidopsis**. Conserved domains of RPD3/HDA1 histone deacetylases in *Arabidopsis thaliana *are shown with their corresponding amino acid length on the right. Arrows indicate histidine active site in histone deacetylase (HD) domain. Abbreviations and amino acid residues in parenthesis: PB, Pfam B database; DUF, domain of unknown function; G, poly-glycine rich region (HDA6: 311–314, HDA7: 302–305, HDA9: 296–299, HDA17: 27–31); Asp, aspartate rich region (HDA6: 428–465, HDA9: 384–424, HDA10: 100–140, HDA17: 116–156, HDA15: 58–77); BAD, BH3-only pro-apoptotic domain; ad, aldehyde dehydrogenase (HDA15: 25–36); ZF, Zinc finger (86–115); 60s RP, ribosomal protein (193–203); CR6, cytokine-responsive protein 6 interacting protein (402–559); LZ, leucine zip motif (457–478); CC, coiled-coil domain (430–610); COG6, conserved oligomeric golgi complex 6 (629–641); RtcR, RNA terminal phosphate cyclase regulator (297–312); NLS, nuclear localization signal (HDA6:17–20 and 14–20, HDA19: 12–18 and 428–434, HDA5: 10–26, HDA14: 406–412, HDA15: 14–17, 521–537 and 522–538, HDA18: 43–59 and 121–137); NES, nuclear export signal (HDA2, 180–187 and 245–257, HDA8: 162–168, HDA15: 484–491, HDA18: 538–549).

Different databases such as Pfam [21], InterPro [22], Automatic Domain Decomposition Algorithm [23], and UniProtKB/TrEMBL [24] were used to determine the conserved domains of these histone deacetylases (Figure 4). Based on the sequence analysis from Pfam database, a new conserved domain was detected in AtHDA9. A BH3-only pro-apoptotic domain (BAD) from the Bcl-2 protein family is encoded at amino acids 385–402. This protein family is highly regulated by phosphorylation in response to survival factors [25] not to mention phosphorylation-regulated 14-3-3 binding with BAD proteins [26]. Although AtHDA10 and AtHDA17 are classified under the RPD3 group due to sequence similarity with their histone deacetylase domain, they do not contain any active sites.

On the other hand, Class II HDAs manifest variable conserved domains. AtHDA5 contains the histone deacetylase conserved region at amino acids 46–348 with a histidine active site located at amino acid 158. Two other conserved regions were identified by Pfam-B at amino acids 383–436 (Pfam-B 134244) and 438–659 (Pfam-B 18135). As retrieved from the Automatic Domain Decomposition Algorithm (ADDA) database, Pfam-B 134244 corresponds to a phosphate ABC transporter which is a substrate binding component. On the other hand, Pfam-B 18135 corresponds to a conserved 117 amino acid, hypothetical protein found in *Pyrococcus horikoshii*. Both Pfam-B regions were also found in its monocot orthologues in maize MzHDA109 and rice OsHDA713 and OsHDA2213. The poplar orthologue, PtHDA906, on the other hand, also contains the Pfam-B 18135 region together with a conserved sedlin N and transposase 24 domain. Sedlin N functions in endoplasmic reticulum-to-golgi transport while transposase 24 are necessary for efficient DNA transposition.

AtHDA8 contains a histidine active site at amino acid 145 in the histone deacetylase domain (amino acids 39–335). The conserved histone deacetylase region in AtHDA14 was estimated at amino acids 80–387 with an active site at 202 (H). In addition, AtHDA14 also contains a predicted integral membrane protein DUF2275 which is found in various hypothetical bacterial proteins and in RNA polymerase sigma factor. However, its relevant function in AtHDA14 remains to be elucidated. Its plant orthologues also contain domains which can be traced back to prokaryotic proteins but with unknown function such as DUF1820 in PtHDA901 and DUF2089 in PpHDA1509. However, its monocot orthologues uniquely display variable conserved regions such as Rab5-binding domain for both rice orthologues, OsHDA714 and OsHDA2214. This domain allows binding to GTPase Rab5 necessary for Rab5-dependent recruitment of Rabaptin5 to early endosomal membranes [27]. The maize orthologue, MzHDA118, on the other hand, contains RanBP1 which is known to regulate receptor mediated transport between nucleus and cytoplasm.

In AtHDA15, the conserved histone deacetylase domain was estimated at amino acids 171–461 with its active H site at amino acid 277. AtHDA15 contains a zinc finger RanBP2 type region encoded at amino acids 86–115. This Zn finger RanBP2 domain also appears to be conserved in its lower plant orthologue, PpHDA1501 and PpHDA1510, and monocot orthologues in rice, OsHDA704, and maize, MzHDA110. RanBP2 has been well implicated in nucleocytoplasmic transport. Its zinc finger has a high binding specificity to exportin-1 (CRM1), a nuclear export factor, serving as its docking site for nuclear export [28]. Moreover, it also contains an uncharacterized DUF2086 protein which is conserved in bacteria. Based on the Pfam B database, an overlapping domain, Pfam-B 108730, is encrypted at amino acids 1–109, which is also found in its maize orthologue, MzHDA110, at amino acids 24–82 and poplar, PtHDA907, at amino acids 28–135. This uncharacterized Pfam-B domain is homologous to a cDNA, FLJ32790 FIS, found in humans and in pufferfish, *Tetraodon nigroviridis*, which encodes a Tau-tubulin kinase 2 belonging to a CK1 Ser/Thr protein kinase family wherein mutations or defects of which causes spinocerebrallar ataxia. This coincides with the InterPro signature database classifying this region as a short repeat motif that will not fold into a globular domain on its own unless more copies are present. Tau-tubulin proteins generally promote microtubule assembly and stabilize microtubules. Thus, it is possible that AtHDA15 may localize and function not only in the cytoplasm but also in the cytoskeleton and plasma membrane as well. In addition, an aspartate-rich region was detected by UniProtKB at amino acid 58–77. The monocot orthologue of AtHDA15, ZmHDA110, contains a zinc finger C3HC4 type RING finger that plays a key role in ubiquitination pathway.

AtHDA18 is a tandem duplication of AtHDA5 with mutational insertions and deletions including an additional α helical domain near its C terminal region. Aside from a coiled-coil domain and leucine zip motif, based on the pfam database, AtHDA18 also contains a domain of unknown function, DUF220, at amino acids 43–55 which can be traced back to archea and eubacteria. It also contains a 60s acidic ribosomal protein encoded at amino acids 193–203 which serves a structural component of ribosome for translational elongation. At amino acids 402–559, a CR6 interaction protein is encrypted which is known to be involved in growth arrest and DNA-damage inducible protein interaction. CR6 interaction protein act as negative regulators of G1 and S cell cycle phase progression by inhibiting cyclin-dependent kinases. They also function as a repressor of orphan nuclear receptor, NR4A1, by inhibiting AB domain-mediated transcriptional activity [29]. Also, a conserved oligomeric complex, COG6, domain is found at amino acids 629–641. COG6 is a component of a conserved oligomeric golgi complex and is required for normal golgi morphology and localization [30,31]. There are other 246 Pfam-B matches for AtHDA18 which still remains to be described and annotated.

For Class IV, AtHDA2 contains a domain of unknown function, DUF1409, at amino acids 75–86 generally described as short conserved sequences found mostly in hypothetical *Oryza sativa *proteins with unknown function. Furthermore, a regulator of RNA terminal phosphate cyclase, RtcR, is encrypted at amino acids 297–312. This is usually found at the central region of protein sequences and is known as a sigma54-dependent enhancer binding protein which activates the transcription of the rtcBA operon [32].

### Subcellular Localization

Bioinformatics data were generated from five different programs to predict the possible localization sites of Class II & IV HDAs (Table 3). Forecasts from TAIR were limited only to AtHDA5 and AtHDA14 with no localization predictions to AtHDA2, AtHDA8, AtHDA15, or AtHDA18. The Subcellular Localization of Proteins using Local alignment (SLP-L) program [33], on the other hand, generated very low reliability scores in the predicted subcellular localization of Class II and Class IV histone deacetylases. Similarly, WoLF PSORT [34] reported low observed frequency values for both classes of HDAs. However, AtHDA5 scored relatively high for both nuclear and cytoplasmic localizations suggesting a possibility for nucleo-cytoplasmic transport. NetNES predicts nuclear export signals (NES) in AtHDA2, AtHDA8, AtHDA15, and AtHDA18 with a threshold value of 0.5. Surrounding amino acids of predicted NES residues also exhibit low intensity signals below the set threshold value. Based on sequence analyses of PSORT II, AtHDA5, AtHDA14, AtHDA15, and AtHDA18 contain nuclear localization signals (NLS) which follow a bipartite, pattern 7, or pattern 4 type NLS.

**Table 3:** Subcellular localization of Class II and Class IV histone deacetylases in Arabidopsis were predicted using different databases and programs.

**HDA**	**TAIR**	**SLP-L^A^** (Reliability index)	**WoLF PSORT^B^**	**NetNES^C^** (predicted NES)	**PSORT II** (NLS score)^D^
**AtHDA2**	---	mitochondria (1)	cytosol (7) nucleus (4) chloroplast (1) mitochondria (1)	182-I 252-L 254-V	--- --- -0.47

**AtHDA5**	nucleus & cytosol	nucleus or cytosol (2)	nucleus (6.5) cytosol & nucleus (6.5) cytosol (5.5)	---	10–26 aa bipartite NLS 0.02

**AtHDA8**	---	nucleus or cytosol (2)	cytosol (10) cytoskeleton(2) nucleus (1)	168-L	--- --- -0.47

**AtHDA14**	chloroplast	chloroplast (1)	chloroplast (13)	---	406–412 aa par7 NLS -0.13

**AtHDA15**	---	nucleus or cytosol (3)	cytosol (8) nucleus (2) chloroplast (1) plastids (1) peroxisomes (1)	489-I	14–17 par4 521–537 bipartite 522–538 bipartite 0.70

**AtHDA18**	---	nucleus or cytosol (1)	cytosol (6) nucleus (3) chloroplast (2) plastids (2)	541-L	43–59 bipartite 121–137 bipartite 0.51

^A ^Reliability index ranges from 1 to 10. As the RI increases, the prediction result becomes more reliable.

^B ^The predictions of WoLF PSORT is based on the protein's amino acid sequence identifying target signals from proteins in the dataset which truly localize in the area. The numbers in parenthesis indicate the prior probability that such protein localizes to a given site is equal to the proportion of proteins in WoLF PSORT's dataset which is 456 (nucleus), 432 (cytosol), 750 (chloroplast), 210 (mitochondria), 11 (cytosol & nucleus), 41 (cytoskeleton), 165 (plastids), and 52 (peroxisomes).

^C ^NetNES results indicate predicted nuclear export signal (NES) with the corresponding amino acid number and residue. A NetNES score of 0.5 was set as a threshold for positive NES prediction although residues surrounding the predicted NES usually exhibit signal intensities below the set threshold value.

^D ^Numbers reflect amino acid residues exhibiting nuclear localization signals (NLS). Types of NLS detected from Class II were either bipartite or the classical type, pat4 or pat7, derived from SV40 large T antigen. Pattern for bipartite NLS follows 2 basic K/R residues, 10 residue spacer, and another basic region composed of at least 3 K/R residues out of 5 amino acids. Pattern 4 (pat4) is composed of 4 basic residues (K or R) or 3 basic residues and either H or P. Pattern 7 (pat7) starts with P then followed within 3 residues by a basic segment with 3 K/R residues out of 4. NLS score delineates the tendency of the protein to be either nuclear or cytoplasmic which is calculated based on its amino acid composition according to the neural network developed by Reinhardt & Hubbard (1998). Positive numbers indicate higher probability for nuclear localization whereas negative numbers lean towards more cytoplasmic localization.

### Expression Patterns of RPD3/HDA1 family Histone Deacetylases

**Figure 5:**
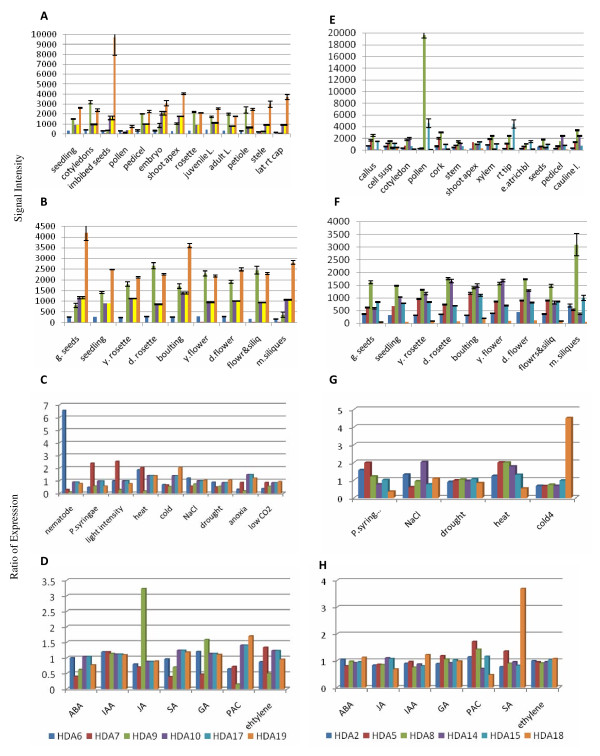
**Expression patterns of RPD3/HDA1 histone deacetylases based on Genevestigator**. Expression patterns of Class I (A-D), Class II and Class IV (E-H) histone deacetylases are shown in different anatomical parts (A, E), developmental stages (B, F), and those induced by biotic and abiotic stress (C, G), and hormones (D, H). These data have been generated using Genevestigator produced from microarray data by Schmidt *et al. *(2005) and Kilian *et al. *(2007).

Based on the Genevestigator from the microarray data generated by Schmidt *et al *[35] and Kilian *et al. *[36], AtHDA19 and AtHDA9 expressions significantly predominate other class I histone deacetylases in all the vegetative parts as well as developmental stages of the plant (Figure 5). The highest expression for AtHDA19 can be observed at the germinating seeds and bolting stages as well as imbibed seeds. In addition, cold stress significantly induces its expression. AtHDA9 is at its peak during developed rosette stage and concentrate mostly in the cotyledons. Elevated expression of AtHDA9 can further be enhanced by JA and GA treatment. It can be observed that AtHDA6 appears equally expressed in all the plant parts all throughout the developmental stages but is highly modulated by biotic stress induced by nematode. On the contrary, AtHDA7 do not seem to be expressed at all in any parts of the plant nor during developmental stages. However, its expression can be induced by ethylene, light intensity, heat, and biotic stress by *Pseudomonas syringae*. The expression patterns of AtHDA10 and AtHDA17 are similar in all the plant parts in all the developmental stages and consistently the same still in response to all the stress treatments.

On the other hand, Class II histone deacetylases have varied expression patterns with AtHDA8 predominating mostly all developmental stages with its peak of expression during mature silique stage and in pollen and cauline leaf parts. AtHDA14 manifests its expression starting at the seedling stage with its highest during young flower. In addition, AtHDA15 is significantly expressed at the root tip and pollen while AtHDA5 is at its highest during boulting stage and in cork and xylem. Although AtHDA18 appears to be at a minimum during developmental stages and organ parts, its expression can be induced by IAA, SA, and cold treatment. Moreover, heat stress seems to upregulate most of the Class II HDAs while NaCl treatment only stimulates AtHDA14 and AtHDA2 expression. Furthermore, the expression levels of all these genes, especially AtHDA5 and AtHDA2 are somehow affected by biotic stress induced specifically by *Pseudomonas syringae*.

## Discussion

With the exception of AtHDA10 and AtHDA17, all the RPD3/HDA1 family histone deacetylases studied contain histidine as an active site. Active sites are usually known as catalytic pockets of enzymes where a substrate is bound and converted to a product which is eventually released. In this case, acetyl groups are released. Thus, since most of the RPD3/HDA1 histone deacetylases contain histidine as an active site, it can then be speculated that all these HDACs are functional as a histone deacetylase. However, mutational inactivation studies such as converting histidine to tyrosine still needs to be conducted to confirm the functionality of these active sites. Although HDA10 and HDA17 both contain the conserved RPD3 histone deacetylase domain, they may not be enzymatically functional due to the absence of an active site. These two have been speculated to be mere gene fragments but its identical expression in the microarray data may somehow suggest subfunctionalization. However, there is extensive evidence for HDAC-HDAC interaction in humans where the RPD3-like HsHDAC501 and HsHDAC502 were found in the same multiprotein complex [37]. In HsHDAC510, the C-terminal catalytic domain lacks an active pocket required to activate its enzymatic activity. Its deletion, however, led to its sensitivity to histone deacetylase inhibitors, trapoxin and sodium butyrate, suggesting that its functional N-terminal and inactive C-terminal catalytic domains interact [38]. Thus, we cannot exclude the possibility that AtHDA10 and AtHDA17 may interact and be functional with an active histone deacetylase.

Domain analysis indicated that AtHDA6, AtHDA7 and AtHDA9 have a poly-glycine rich region in the histone deacetylase domain. In addition, both AtHDA6 and AtHDA9 have an aspartate rich region at the C terminus. Furthermore, AtHDA9 contains a BH3-only pro-apoptotic domain (BAD) which is known to bind with 14-3-3, thus, may undergo nucleo-cytoplasmic transport. The specific domains identified in different class I HDACs suggest functional specification. Indeed, AtHDA6 appeared to be unique among the class I HDACs in Arabidopsis since it is the only one that was found to be important in controlling epigenetic states such as DNA and histone methylation [10-13], although the molecular basis for how AtHDA6 is involved in these process is still unclear.

There is extensive evidence to show that plant histone deacetylases act as global transcriptional regulators playing crucial roles in a range of plant developmental processes and plant responses to a variety of environmental stresses [3-9]. AtHDA19 has been well studied as a global repressor where 7% of the plant's genome is either up- or down-regulated in *hda19 *mutants [5]. Furthermore, mutant lines exhibited a wide range of developmental abnormalities affecting flowers and siliques, premature death of seedlings, reduced male & female fertility, and embryonic defects [3,4,8]. AtHDA19 has also been shown to work antagonistically with GCN5 to regulate light-mediated processes [6]. Upon biotic stress such as wounding and pathogen infection, it regulates gene expression in JA and ethylene signaling pathways [16]. In a recent study by Kim *et al *[9], it has been demonstrated that AtHDA19 regulates plant basal response via interaction with WRKY transcription factors. Similar to AtHDA19, AtHDA6 is also known as a global repressor involved in JA pathway, senescence, flowering, and repression of embryonic properties [8,10-14].

Prior studies by Pandey *et al*. [1] on the sequence analysis of AtHDA8 and AtHDA14 suggested *S. cerevisiae *HDAC protein Hos3 as the closest related protein but with very low bootstrap support. Thus, with the failure to identify more closely related proteins to HDA8 and HDA14, other than Hos3, they have concluded AtHDA8 and AtHDA14 to be mere relatives of Class II proteins with AtHDA8 seemingly more related to the prokaryotic acetylpolyamine aminohydrolase proteins than Class II. With the recent updates of databases, we have data mined and analyzed the possible orthologues of these 2 proteins in both lower and higher plants establishing its classification as a Class II histone deacetylase. Moreover, the formation of three clusters separating the AtHDA8 group, AtHDA14 group, and the originally identified class II genes, AtHDA5, AtHDA15, and AtHDA18 may suggest functional diversification.

With AtHDA15 containing a RanBP2 type zinc finger which serves as a docking site for exportin-1 for nuclear export, it also contains a Tau-tubulin kinase 2 which promotes microtubule assembly and stabilization which oftentimes result in a stiffening effect of the microtubules. Thus, this may append the potential of AtHDA15 to localize not only in the cytoplasm but aggregate as well in microtubules, cytoskeleton and plasma membrane. On the other hand, AtHDA18 may play multiple roles other than histone deacetylation and transcriptional repression. With its multiple conserved domains intact, it is probable that HDA18 may also play crucial roles in translational elongation, cell cycle inhibition, DNA-damage mechanisms, and golgi morphology. Although its amino acid sequence is merely a product of duplication of AtHDA5 coupled with evolutionary winnowing, AtHDA18 appears to be an interesting protein among the Class II HDAs due to its multiple conserved domains and upregulation by stress treatments. However, functional analyses such as loss-of-function and/or gain-of-function studies as well as localization analysis are needed to further analyze its potential functions.

AtHDA18 has been implicated to have an active role in root epidermal patterning such that reduced HDAC activity via trichostatin A (TSA) treatment deregulates the expression of key patterning genes *GLABRA2 *(*GL2*), *CAPRICE *(*CPC*) and *WEREWOLF *(*WER*) [17]. Such deregulation had led to elevated expression of *CPC *and *GL2 *at the hair forming (H) position leading to increased root hair formation and altered cellular patterning in *hda18 *mutants. Although *hda5 *and *hda14 *mutants similarly exhibited increased root hairs, only HDA18 was implicated to be involved in root epidermal patterning due to its altered cellular pattern similarly manifested by TSA treated plants. However, it still remains unclear how HDA18 specifically takes part at the interplay between these key genes in root epidermal patterning and the yet unidentified "positional cue". In a recent study conducted by Caro *et al *[39], a *GL2*-expression modulator (GEM) is recruited specifically at the promoter regions of CPC and GL2 manifesting histone H3 hyperacetylation and H3K9 methylation restricting cell division and negatively regulating *GL2 *expression. Assuming the CR6 domain in AtHDA18 is functional, there is a possibility for AtHDA18 acting in tandem with GEM maintaining a closed chromatin configuration at the *GL2, CPC*, and/or *WER *loci while simultaneously modulating cell division by inhibiting cyclin dependent kinases for DNA licensing. In another perspective, there is a possibility for functional redundancy between HDA5 and HDA18, since HDA18 is basically a duplication of HDA5 and share 84% homology mostly in the conserved HDA domain [1].

**Figure 6:**
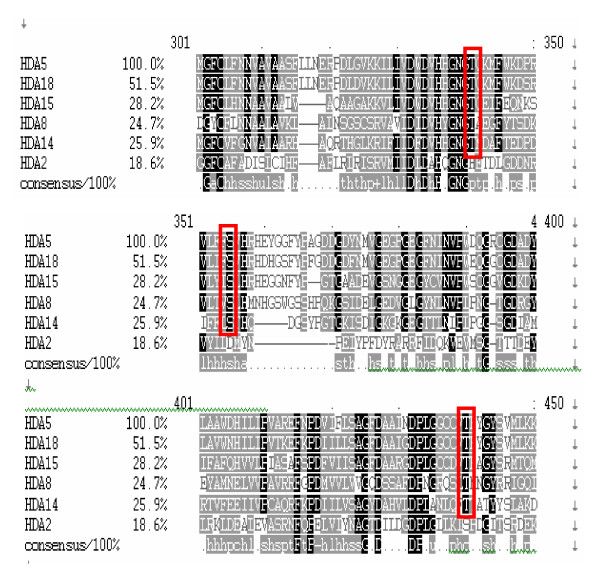
**Multiple sequence alignment of Class II and Class IV histone deacetylases in Arabidopsis**. Multiple sequence alignment of Class II and Class IV histone deacetylases was generated using ClustalW. Three putative conserved serine/threonine residues (red box) may be potential binding sites for 14-3-3 proteins for nucleocytoplasmic transport.

Most of the plant Class II HDACs contain nuclear export and import signals indicating their potential for nucleocytoplasmic shuttling. Similar to mammalian Class II HDACs, all 5 plant Class II HDACs contain conserved Ser/Thr residues (Figure 6) which are potential phosphorylation sites for 14-3-3 binding. However, mammalian Class II HDACs are group into Class IIa (HsHDA504, HsHDA505, HsHDA507, HsHDA509) and Class IIb (HsHDA506 and HsHDA507) [20]. Class IIa HDACs are dependent on 14-3-3 binding to translocate into the cytoplasm and binds with myocyte enhance factor 2 (MEF2) when it shuttles back to the nucleus and becomes active as a histone deacetylase. On the other hand, Class IIb HDACs contain double domains and are dependent on strong NES and NLS for its nuclear import and export. In plants, however, all the Class II HDAs may potentially be regulated via 14-3-3 proteins for cytoplasmic translocation. Given the proper signals for these HDAs to be translocated back into the nucleus, dephosphorylated, and activated as a histone deacetylase, they may bind to MADS-box type II transcription factors which are the plant homologues of MEF2 [40].

In addition, AtHDA2 is still neither classified as Class I nor Class II but rather confirms Pandey *et al *[1] findings isolating it as a separate group within the RPD3/HDA1 superfamily. To refrain from ambiguous and overlapping class groupings, AtHDA2 and its plant orthologues have been classified as Class IV since AtHDA2 is the plant orthologue of the mammalian HsHDA511 previously designated as Class IV [2,41]. Although we found its corresponding orthologues from all the six plants studied, it still remains unclear how this protein differs from Class I and Class II other than functional diversification. In terms of localization based on bioinformatics, AtHDA2 may similarly be exported out of the nucleus just like Class II HDAs due to the presence of multiple NES. However, the mechanisms underlying such process may be different from Class II. With the rtcR imprinted within its sequence, it is more likely that AtHDA2 remains nuclear as it may be actively involved in regulating the transcription of the rtcBA operon. Nevertheless, further experiments should be conducted to confirm its localization site/s as well as functional activities within and outside the nucleus.

It should be noted, however, that all the Class II and Class IV histone deacetylases still contain conserved domains with unknown functions which can be traced back from prokaryotic origins. Based on its evolutionary history, histone deacetylases belong to an ancient protein superfamily which also includes acetoin ulitilization proteins and acetylpolyamine amidohydrolases found in eubacteria and archeal bacteria, respectively. Thus, the evolutionary retention of these domains suggests critical functions which may or may not influence its role as a histone deacetylase.

## Conclusion

The RPD3/HDA1 histone deacetylase family in plants is divided into three distinct groups namely, Class I, Class II, and Class IV. All the 62 genes studied from six different plant species were grouped accordingly identifying their corresponding orthologues. Moreover, Class II comprises not only AtHDA5, AtHDA15, and AtHDA18 but also includes AtHDA8 and AtHDA14 with their parallel orthologues from all the six plants exhibiting similar conserved domains. Although there is a high prognosis for this group to translocate both in the nucleus and cytoplasm due to the presence of NES, NLS, and conserved Ser/Thr residues for 14-3-3 binding, intensive studies are needed to further support this. If plant Class II HDAs can indeed translocate into the cytoplasm, nucleocytoplasmic shuttling then becomes a hallmark for all Class II histone deacetylases likely conserved in the eukaryotes. Thus, it is speculated that this novel characteristic trait may be well encrypted within its conserved regions which is part and parcel of its versatile function and regulation as a histone deacetylase. In addition, there are numerous conserved domains imprinted within each RPD3/HDA1 protein indicating versatile functions other than histone deacetylation and transcriptional repression but also in phosphate ABC transport, microtubule assembly, and golgi morphology just to name a few. Nevertheless, further studies are still needed to elucidate clearly the functional roles of these histone deacetylases in plants.

## Methods

### Multiple sequence alignment

Nucleotide sequences of RPD3/HDA1 histone deacetylases were sequenced and reconfirmed using the TAIR database . Amino acid and nucleotide sequences in fasta formats retrieved from the plant Chromatin Database (ChromDB)  were also used for multiple sequence alignment using ClustalW2 . Highly conserved regions were highlighted in black. Generated phylograms based on the sequence alignments from ClustalW2 were compared with phylogenetic trees inferred from MEGA4.

### Bioinformatics analyses

Amino acid sequences in fasta formats were analyzed using different databases such as Pfam , InterPro Scan , Automatic Domain Decomposition Algorithm , and UniProtKB/TrEMBL  to further explore the conserved domains of Class II histone deacetylases. In addition, subcellular localization of each of the 6 proteins was predicted using TAIR , SLP-L , and WoLF PSORT . Nuclear export signals were detected using NetNES [42] while nuclear localization signals were inferred using PSORTII [43].

### Phylogenetic analyses

Amino acid as well as nucleotide sequences in fasta format were retrieved from ChromDB. These were further configured into a fasta format based on its conserved domain using the MAFFT program at EMBL-EBI [44] prior to analysis. MAFFT fasta formatted sequences were keyed in for phylogenetic analyses using the Molecular Evolutionary Genetic Analysis 4 (MEGA4) program [45]. Tree reconstruction was inferred using the Neighbor Joining method with a Poisson correction model and a bootstrap test of 1000 replicates.

### Analyses of expression patterns using Genevestigator

RPD3/HDA1 genes were individually queried for their expression patterns using Genevestigator [46]. Only highly pertinent expression levels in different anatomical parts, hormone inducers, as well as biotic and abiotic stresses were included. However, the expression levels of all the RPD3/HDA1 in all the developmental stages were considered in the study.

## Authors' contributions

MVA carried out the phylogenetic and bioinformatics studies, conducted analysis for expression patterns of Class II and Class IV HDAs, and drafted the manuscript. CWY conducted analysis for expression patterns of Class I HDAs and help draft the manuscript. KW conceived and designed the study and drafted the manuscript. All authors read and approved the final manuscript.
